# Aberrant lncRNA expression in patients with proliferative diabetic retinopathy: preliminary results from a single-center observational study

**DOI:** 10.1186/s12886-023-02817-4

**Published:** 2023-03-10

**Authors:** Lan Zeng, Minwen Zhou, Xiaocong Wang, Xiaofeng Long, Meng Ye, Yuan Yuan, Wei Tan

**Affiliations:** 1 Zunyi Medical University, Zunyi, Guizhou China; 2 Department of Ophthalmology, The Third Affiliated Hospital of Zunyi Medical University (The First People’s Hospital of Zunyi), Zunyi, Guizhou China; 3 Department of Ophthalmology, Shanghai General Hospital, Shanghai, China

**Keywords:** Proliferative diabetic retinopathy, Long noncoding RNA, Vitreous, Anti-vascular endothelial growth factor

## Abstract

**Background:**

Diabetic retinopathy (DR) is a leading cause of blindness. Vision threat is particularly severe in patients with retinal neovascularization. However, little is known about the role of long noncoding RNAs (lncRNAs) in proliferative diabetic retinopathy (PDR). The goal of this study was to identify lncRNAs involved in PDR.

**Methods:**

We compared lncRNA expression profiles in the vitreous between patients with PDR and those with idiopathic macular hole (IMH) and between patients with PDR who had received anti-vascular endothelial growth factor (VEGF) therapy and those who had not. Vitreous samples from patients with PDR and IMH were screened for lncRNAs using microarray-based analysis, and quantitative real-time polymerase chain reaction (qRT-PCR) was used to confirm the microarray results. Bioinformatic analysis was also performed. Moreover, the effect of anti-VEGF therapy was investigated in vitreous samples of patients with PDR treated with anti-VEGF therapy and those who were not.

**Results:**

A total of 1067 differentially expressed noncoding RNA transcripts were found during screening in the vitreous humor of patients with PDR than in those with IMH. Five lncRNAs were subjected to qRT-PCR. RP11-573 J24.1, RP11-787B4.2, RP11-654G14.1, RP11-2A4.3, and RP11-502I4.3 were significantly downregulated; this was validated by the comparison using the microarray data. In addition, 835 differentially expressed noncoding RNA transcripts were found during screening in the vitreous humor of patients with PDR treated with anti-VEGF therapy compared with untreated PDR patients. RP4-631H13.2 was significantly upregulated, which is consistent with the trend of the microarray analysis.

**Conclusions:**

There were systemic expression differences in the vitreous at the microarray level between patients with PDR and those with IMH and between patients with PDR after anti-VEGF treatment and those that did not receive anti-VEGF treatment. LncRNAs identified in the vitreous humor may be a novel research field for PDR.

**Supplementary Information:**

The online version contains supplementary material available at 10.1186/s12886-023-02817-4.

## Background

Diabetic retinopathy (DR) is the most common complication of diabetes mellitus and the leading cause of blindness in middle-aged and elderly individuals [1, 2]. It is classified as non-proliferative diabetic retinopathy (PDR) and PDR [3]. Retinal neovascularization is a key feature of PDR [4]. The main therapeutic strategy is the use of anti-vascular endothelial growth factor (VEGF) [5]. However, resistance to neovascularization remains challenging owing to its ineffectiveness.

Long noncoding RNAs (lncRNAs) are non-protein-coding transcripts larger than 200 nucleotides. They can participate in gene regulation at transcriptional, post-transcriptional, and translational, such as regulatory transcription factors and endogenous competitive RNA [6]. LncRNAs have been implicated in a wide range of physiological processes and in the pathophysiology of several diseases, and DR is no exception. They are increasingly being recognized as important players in the development of DR.

Aberrant Expression of lncRNAs in PDR may be relevant to the molecular etiology of DR. Some researchers performed microarray-based gene expression analysis designed to serve as a resource for elucidating lncRNA-mediated DR pathogenesis [7, 8]. The identification of dysregulated lncRNAs is a key step in understanding the significance of lncRNAs in DR. The vitreous humor of patients with PDR can represent a reservoir of pathological signaling molecules because of tissue accessibility [9]. The lncRNAs that are differentially expressed in the vitreous humor of patients with PDR remain inadequately explored [9]. Therefore, this is a novel research field for PDR.

In addition, it is necessary to identify lncRNA expression profiles in the vitreous fluid of patients with PDR after anti-VEGF therapy. Adjuvant intravitreal injection of anti-VEGF drugs before vitrectomy, which reduces the difficulty of surgery and recurrence of vitreous hemorrhage, is beneficial [10]. Nevertheless, in some cases, the patient’s eyes fail to respond adequately. Thus, a complete understanding of the relationship between lncRNAs and anti-VEGF drugs may enable add another dimension to its therapeutic directions [11].

In this study, we aimed to detect differences in the expression of lncRNAs and messenger RNAs (mRNAs) between patients with PDR and those with idiopathic macular hole (IMH) and between patients with PDR treated with anti-VEGF therapy and untreated patients with PDR. To reveal the functional significance of lncRNAs in DR, we performed bioinformatic analysis.

## Methods

### Patient recruitment

This clinical study adhered to the provisions of the Declaration of Helsinki for research involving human subjects. This study was approved by the Ethical Review Committee of the First People’s Hospital of Zunyi (Zunyi, China; project number 2019–019). All patients who underwent pars plana vitrectomy surgery for IMH or PDR at the First People’s Hospital of Zunyi between October 2019 and September 2020 were enrolled consecutively.

The patients were divided into three groups: group A consisted of patients with IMH without diabetes, group B consisted of patients with PDR pretreated with conbercept 3–7 days before surgery, and group C consisted of patients with PDR who underwent surgery alone. All subjects underwent a complete ophthalmologic examination, including medical history, best-corrected visual acuity measurement, intraocular pressure measurement, slit-lamp examination, fundus examination, ocular ultrasonography, optical coherence tomography, and fundus fluorescein angiography (as necessary). According to the international classification, PDR is defined as neovascularization and/or vitreous/preretinal hemorrhage [3].

Subjects with other systemic diseases such as renal failure or malignant tumors were excluded. Subjects with other eye diseases, including glaucoma, uveitis, age-related macular degeneration, retinal artery occlusion, retinal vein occlusion, rhegmatogenous retinal detachment, endophthalmitis, or ocular trauma, were also excluded. Patients who had undergone one or more of the following: eye surgery, retinal laser photocoagulation, intravitreal steroids, or anti-VEGF therapy (only 3–7 days before surgery were allowed) were excluded from the analysis. Also, subjects were excluded if they showed any evidence of systemic or local inflammation within 6 months.

Finally, three patient samples from each group were analyzed using microarray technology. The remaining 39 patients (6 in group A, 8 in group B, and 25 in group C) were included in the confirmation cohort. There were no statistically significant differences in the age or sex ratios (Table 1).

**Table 1:** Clinical characteristics of the enrolled patients

Cohort	Age (y), mean ± SD	Female, n (%)	hemoglobin A1c (%), mean ± SD
Screening Cohort			
Group A (*n* = 3)	55.67 ± 12.34	2 (66.67)	4.90 ± 0.40
Group B (*n* = 3)	57.67 ± 3.06	2 (66.67)	10.43 ± 2.49
Group C (*n* = 3)	58.67 ± 8.02	2 (66.67)	9.17 ± 2.20
*P*	0.91	1.00	0.02
PDR Confirmation Cohort			
Group C (*n* = 11)	56.55 ± 9.86	7 (63.64)	8.26 ± 1.30
Croup A (*n* = 6)	57.33 ± 10.50	5 (83.33)	4.90 ± 0.45
*P*	0.88	0.60	< 0.01
Anti-VEGF Confirmation Cohort			
Group B (*n* = 8)	49.00 ± 19.54	5 (62.50)	10.26 ± 4.04
Croup C (*n* = 14)	58.07 ± 8.72	7 (50.00)	8.69 ± 2.34
*P*	0.15	0.68	0.34

*P* <  0.05 was considered to be statistically significant

Group A consisted of patients with IMH; Group B consisted of patients with PDR pretreated with conbercept 3–7 days before surgery; Group C consisted of patients with PDR who underwent surgery alone

*PDR* Proliferative diabetic retinopathy, *IMH* Idiopathic macular hole

### Sample processing

A blood sample (approximately 6 mL) was drawn from the forearm vein into a tube containing an anticoagulant following overnight fasting on the day of surgery. The mixture was immediately centrifuged at 4000×g for 10 min at 4 °C. A vitreous sample (approximately 1 mL) was carefully collected into a 2 mL sterile syringe using a 25-gauge vitreous cutter and manual suction before opening the intraocular irrigation system. If vitreous hemorrhage was present, the surgeon avoided collecting blood components as much blood as possible. All samples were stored in cryopreservation tubes and immediately cooled at − 80 °C until analysis.

After sample collection, total RNA was extracted using TRIzol LS reagent (Invitrogen, Carlsbad, CA, USA) combined with miRNeasy Micro Kit (Qiagen, Hilden, Germany). RNA quality and integrity were measured using Nanodrop (Thermo Fisher Scientific, Waltham, MA, USA) and Agilent 4200 TapeStation.

### Microarray analysis

The noncoding RNA and coding RNA transcriptome analysis of the vitreous humor were detected using Clariom D Pico Assay (Affymetrix, Bedford, MA, USA), which has 13,574 transcripts. After assessing RNA quality and quantity, chip analysis was performed by Gminix Biotechnology Company (Shanghai, China). Briefly, sample labeling, hybridization, and washing were performed according to the manufacturer’s protocol. The microarrays were scanned using a GeneChip Scanner 3000 7G (Affymetrix). Raw intensity CEL files generated by GeneChip™ Command Console™ were imported into Transcriptome Analysis Console 4.0.2 (TAC 4.0.2). The data were analyzed with the Robust Multi-chip Analysis algorithm using Affymetrix default analysis setting and global scaling as the normalization method. Quality control graphs were used to assess the quality of sample files. The Limma Bioconductor package (implemented in TAC 4.0.2) was used to analyze expression data based on linear models.

The final difference result was obtained according to the filter condition | fold change | ≥ 1.5 and *P* values < 0.05. Hierarchical clustering was performed to show distinguishable noncoding RNA and coding RNA transcript expression patterns among the samples. All the original data were uploaded to the Gene Expression Omnibus public database (https://www.ncbi.nlm.nih.gov/geo; accession number GSE191210).

### Gene ontology (GO) enrichment analysis and Kyoto encyclopedia of genes and genomes (KEGG) pathway enrichment analysis

The GO database describes our knowledge of the biological domain regarding molecular functions, cellular components, and biological processes. According to the relationship of pathways in the KEGG database, the interaction network of a significant pathway was constructed to find the core pathway that plays a key role [12–14]. Fisher’s exact test was used to select significant GO categories and KEGG pathways, and the significance threshold was defined as *P* values < 0.05.

### Co-expression network

Co-expression networks were constructed to determine the potential roles of noncoding RNA transcripts. To find the co-expression relationship between the differences, Pearson correlation was calculated to identify significantly correlated pairs. It was constructed using Cytoscape, according to the expression value distribution of differential RNA transcripts in different groups. The Pearson correlation value cut-off was = 0.95, with *P* values < 0.05.

### Analysis of neighbor genes of the noncoding RNA transcripts

We searched for noncoding RNA transcripts and their associated coding gene pairs, including the same strand gene with overlap, upstream gene with 10,000 bp, downstream gene with 10,000 bp, and complementary strand gene with overlap.

### Quantitative real-time polymerase chain reaction (qRT-PCR)

To further verify the gene chip results, the threshold for the differential expression of lncRNAs was set to a *P* value < 0.01. Differentially expressed lncRNAs were sorted according to the absolute value of the fold change. Several candidate lncRNAs with multi-variable shear or difficult primer designs were excluded. Five target genes were selected for subsequent qRT-PCR analysis. Total RNA was reverse-transcribed using a PrimeScript RT reagent kit (TaKaRa, Dalian, China), and qRT-PCR was performed using the CFX96 real-time PCR detection system (Bio-Rad, Hercules, CA, USA). Transcript levels were determined using the PCR Master Mix (Solarbio, Beijing, China). The primer pairs used are listed in Table S1. The specificity of the qRT-PCR products was estimated using a dissociation curve. qRT-PCR was performed in duplicate for each sample. The relative gene expression was calculated using the 2^-ΔΔCt^ method. Glyceraldehyde-3-phosphate dehydrogenase (GAPDH) served as the internal control.

### Statistical analysis

All statistical analyses were performed using the SPSS software (version 18.0; SPSS Inc., Chicago, IL, USA). Using the Shapiro–Wilk normality test, the numeric variables were first tested for the normality of distributions. Comparisons between two groups were made using Student’s *t*-test or Mann–Whitney U test. Also, comparisons among three groups were analyzed using a one-way analysis of variance or the Kruskal–Wallis H rank sum test. Categorical variables were determined using Fisher’s exact test. Statistical significance was defined as a *P* value less than 0.05.

## Results

### Evaluation of RNA quality and quantity

The range of extracted RNA from the vitreous sample was 12.0–96.0 ng, and that of the plasma sample was 10.5–87.0 ng. To evaluate RNA quality, A_260_/A_280_ ratio and A_260_/A_230_ ratio were determined. The RNA samples had an A_260_/A_280_ ratio of 1.8 to 2.0, indicating the absence of contaminating proteins. Also, the RNA samples had an A_260_/A_230_ ratio > 2.0, indicating the absence of other organic compounds. We used the RNA integrity number obtained via microfluidic analysis to evaluate RNA integrity. All samples were sufficiently pure and had RNA integrity.

### Transcriptome analysis

**Fig. 1:**
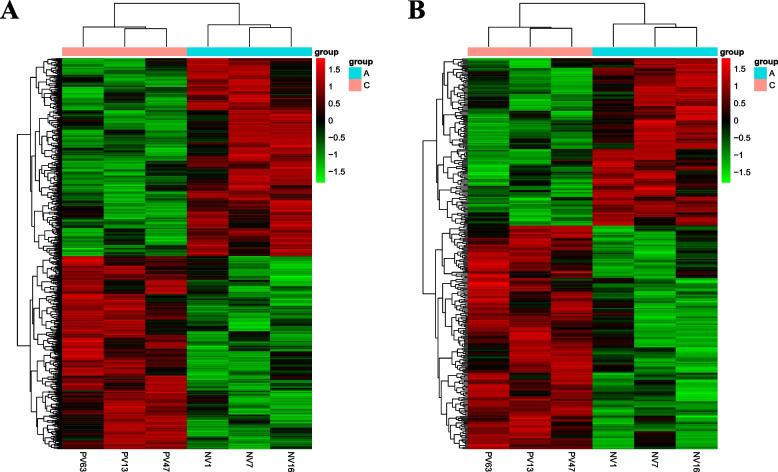
Heatmaps were generated from the hierarchical cluster analysis (Group C versus Group A)

A total of 1067 differentially expressed noncoding RNA transcripts (526 upregulated and 541 downregulated) and 514 differentially expressed coding RNA transcripts (327 upregulated and 187 downregulated) were identified in the vitreous humor of patients with PDR compared with those with IMH. Heatmaps were generated to show the differentially expressed noncoding RNA and coding RNA transcripts (Fig. 1). When anti-VEGF-treated patients with PDR were compared with those untreated, 835 differentially expressed noncoding RNA transcripts (455 upregulated and 380 downregulated) and 226 differentially expressed coding RNA transcripts (124 upregulated and 102 downregulated) were found (Fig. S1).

### Bioinformatics analysis (group C versus group A)

#### GO enrichment analysis

**Fig. 2:**
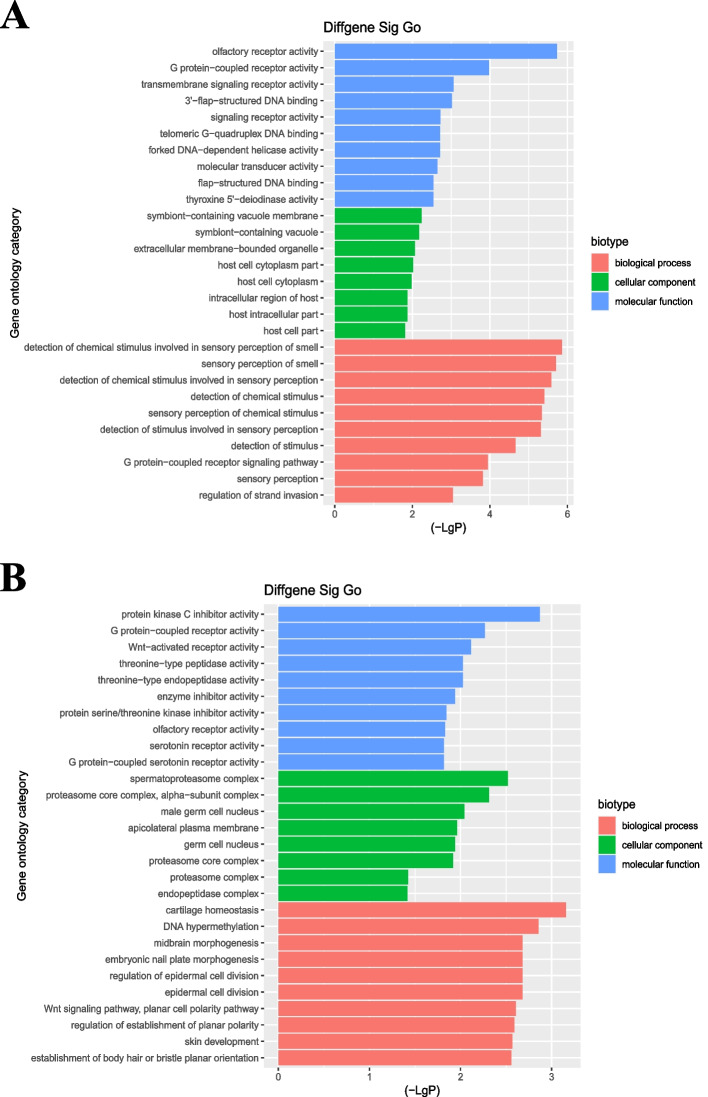
GO Enrichment Analysis (Group C versus Group A)

GO enrichment analysis revealed 113 terms corresponding to upregulated transcripts and 148 terms corresponding to downregulated transcripts. The GOs targets with top enrichment scores [−log10(*P*-value)] by the upregulated transcripts were olfactory receptor activity (ontology; molecular function), symbiont-containing vacuole membrane (ontology; cellular component), and detection of chemical stimulus involved in sensory perception of smell (ontology; biological process) (Fig. 2A). The GOs targets with top enrichment scores for the downregulated transcripts were protein kinase C inhibitor activity (ontology; molecular function), spermatoproteasome complex (ontology; cellular component), and cartilage homeostasis (ontology; biological process) (Fig. 2B).

#### KEGG pathway enrichment analysis

**Fig. 3:**
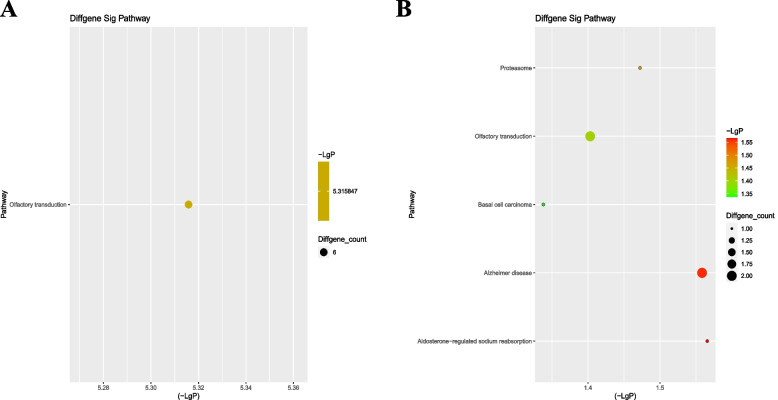
KEGG Pathway Enrichment Analysis (Group C versus Group A)

KEGG pathway enrichment analysis showed that one pathway corresponded to upregulated transcripts, and the enriched pathway was Olfactory transduction (Fig. 3A). Moreover, five pathways corresponded to downregulated transcripts, and the enriched pathways were Aldosterone-regulated sodium reabsorption, Alzheimer disease, Proteasome, Olfactory transduction, and Basal cell carcinoma (Fig. 3B).

#### Co-expression network

**Fig. 4:**
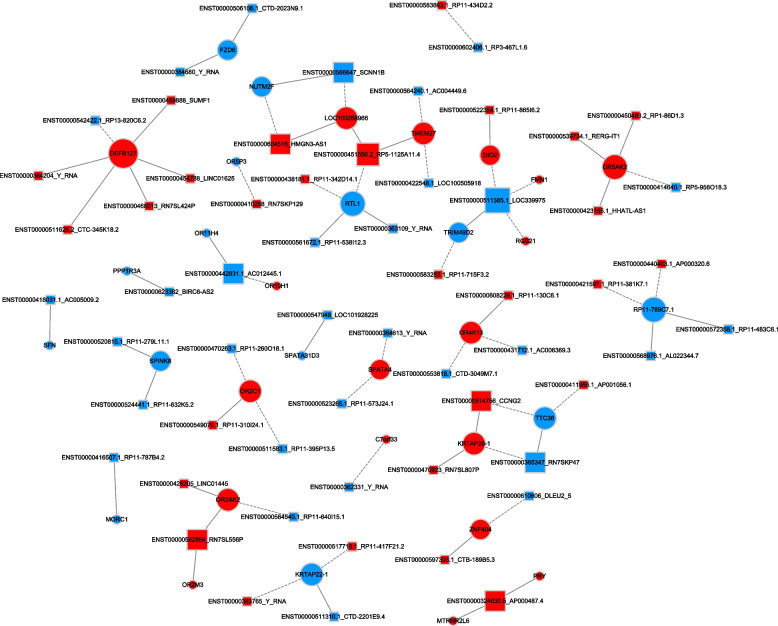
The co-expression network of differential transcripts (Group C versus Group A)

The co-expression network comprised 59 differentially expressed noncoding RNA transcripts and 32 coding RNA transcripts (Fig. 4). The network indicated that coding RNAs could correlate with many target noncoding RNAs and vice versa. The correlation analysis strengthens this argument.

#### Analysis of neighbor genes of the noncoding RNA transcripts

To investigate the possible functions of the noncoding RNA transcripts, we predicted the potential targets of noncoding RNA transcripts. Table 2 contains 39 differentially expressed noncoding RNA transcripts and their associated coding gene pairs.

**Table 2:** Analysis of neighbor genes of the noncoding RNA transcripts (Group C versus Group A)

Gene Symbol	Fold Change	*P*-value	Regulation	Same Strand Gene with Overlap	Up Stream Gene in 10,000 bp	Down Stream Gene in 10,000 bp	Complementary Strand Gene with Overlap
AP000487.4	1.57	0.03	up				PPFIA1
RP11-242O24.3	2.02	0.03	up		SRSF4		
RP5-956O18.3	−2.76	<0.01	down				GALNT2
CITF22-24E5.1	1.76	0.01	up				PVALB
AC005009.2	−1.68	0.01	down				GRM3
RP4-613B23.3	2.03	<0.01	up				HHATL
RP11-49O14.3	−1.98	0.01	down	C9orf3			
AP000320.6	1.8	0.03	up				KCNE2
RP4-631H13.2	−1.89	0.01	down				ZYG11A
RP5-1125A11.4	1.78	0.02	up				RALY
CTD-2023 N9.1	−1.64	0.03	down				ACTBL2
CTD-2116 N20.1	1.92	0.03	up			ADAMTS6	
CTD-2201E9.4	−1.96	0.01	down				SEMA5A
CTC-345 K18.2	1.71	0.04	up				MYOZ3
CTC-348 L14.1	1.58	0.04	up				VCAN
RP11-654G14.1	−2.16	<0.01	down	SLC30A8		SLC30A8	
RP11-279 L11.1	−1.53	0.02	down	CSMD1			
KB-1083B1.1	−1.66	0.03	down				SNX31
RP11-173P15.3	−1.57	0.04	down				MLEC
RP11-1018 J8.2	1.56	0.01	up	RERG	RERG		
RP13-820C6.2	−2.22	0.02	down				EP400
RP11-310I24.1	1.55	0.01	up				TMTC1
RP1-267 L14.3	2.13	0.04	up				NAA25
RP11-344B23.2	−1.67	0.03	down				PAX5
RP11-138H8.6	2.43	0.03	up	LARP6	LARP6		
RP11-133 K1.5	1.71	0.04	up				PLCB2
RP11-538I12.3	−1.73	0.02	down				ADAMTS18
AC004449.6	−1.77	0.04	down		RNF126	POLRMT	
RP11-327F22.5	−1.78	0.04	down		CYLD		
RP11-30 L15.6	1.8	0.01	up		LYPLA1		
RP13-638C3.4	1.6	0.01	up				FOXK2
RP11-227G15.2	1.73	0.03	up		RHBDL3		C17orf75
RP11-715F3.2	1.65	0.02	up	METTL4	METTL4	METTL4	
RP11-640I15.1	−2.17	0.04	down				AC005324.3 TRIM16
RP11-973H7.3	2.84	0.03	up	CEP76	CEP76		PSMG2
CTB-189B5.3	1.58	0.02	up				FBXO27
RP11-91G21.1	−1.54	0.02	down				U2SURP
RP3-467 L1.6	−1.85	0.02	down	VAMP3	CAMTA1	PER3	
LL09NC01-139C3.1	1.58	0.02	up		WDR5		

*P* <  0.05 was considered to be statistically significant

Group A consisted of patients with IMH; Group C consisted of patients with PDR

*IMH* Idiopathic macular hole, *PDR* Proliferative diabetic retinopathy

### Bioinformatics analysis (group B versus group C)

#### GO enrichment analysis

GO enrichment analysis showed that 136 terms corresponded to dysregulated transcripts (109 upregulated and 27 downregulated). The GOs targets with top enrichment scores for the upregulated transcripts were protein-arginine deiminase activity (ontology; molecular function), actin filament (ontology; cellular component), and ureteric bud invasion (ontology; biological process). The GOs targeted with top enrichment scores by the downregulated transcripts were olfactory receptor activity (ontology; molecular function) and detection of chemical stimulus involved in sensory perception of smell (ontology; biological process) (Fig. S2).

#### KEGG pathway enrichment analysis

KEGG pathway enrichment analysis showed that one pathway (Homologous recombination) corresponded to upregulated transcripts, and one pathway (Olfactory transduction) corresponded to downregulated transcripts (Fig. S3).

#### Co-expression network

The co-expression network comprised 11 differentially expressed noncoding RNA transcripts and 10 coding RNA transcripts (Fig. S4).

#### Analysis of neighbor genes of the noncoding RNA transcripts

A total of 32 differentially expressed noncoding RNA transcripts and their associated coding gene pairs are shown in Table S2.

### qRT-PCR

**Fig. 5:**
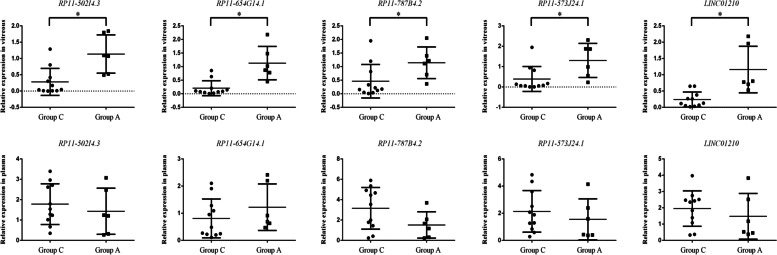
Expression levels of lncRNAs of the confirmation cohort, in vitreous and plasma (Group C versus Group A)

Expression was assessed via individual qRT-PCR assays using vitreous humor and plasma samples from a confirmation cohort. The threshold for the differential expression of lncRNAs was set to a *P* value of < 0.01. All the dysregulated lncRNAs in microarray data analysis are listed in Tables 3 and S3. To verify the results of the microarray analysis independently, five dysregulated lncRNAs were manually selected. The levels of RP11-573 J24.1, RP11-787B4.2, RP11-654G14.1, RP11-2A4.3 (LINC01210 provided by The HUGO Gene Nomenclature Committee symbol), and RP11-502I4.3 were significantly lower in the vitreous humor of patients with PDR than in those with IMH, which was validated entirely by comparing them with microarray data (Fig. 5).

**Table 3:** Dysregulated lncRNAs in microarray data analysis (Group C versus Group A)

Gene ID	Gene Symbol	*P*-value	Fold Change	Regulation
TC0100017689.hg.1	RP5-956O18.3	< 0.01	−2.76	down
TC0X00007085.hg.1	RP11-342D14.1	< 0.01	2.59	up
TC2200008344.hg.1	RP1-231P7P.1	0.01	−2.37	down
TC1500009850.hg.1	RP11-502I4.3	0.01	−2.27	down
TC0300008929.hg.1	RP11-2A4.3	< 0.01	−2.22	down
TC0800008635.hg.1	RP11-654G14.1	< 0.01	−2.16	down
TC1000011831.hg.1	RP11-381 K7.1	0.01	2.16	up
TC0900008542.hg.1	RP11-787B4.2	< 0.01	−2.05	down
TC0300007168.hg.1	RP4-613B23.3	< 0.01	2.03	up
TC0800007818.hg.1	RP11-573 J24.1	< 0.01	−2.00	down
TC0500006707.hg.1	CTD-2201E9.4	0.01	−1.96	down
TC0700011403.hg.1	RP4-736H5.3	< 0.01	−1.92	down
TC2000009883.hg.1	RP5-1009E24.8	< 0.01	−1.91	down
TC0100014213.hg.1	RP4-631H13.2	0.01	−1.89	down
TC0500009510.hg.1	CTB-43E15.1	0.01	−1.88	down
TC1400009841.hg.1	RP11-299 L17.3	0.01	−1.83	down
TC0500013160.hg.1	CTD-2118P12.1	< 0.01	−1.82	down
TC2200007269.hg.1	CITF22-24E5.1	0.01	1.76	up
TC0700011688.hg.1	AC005009.2	0.01	−1.68	down
TC2100007705.hg.1	AP001171.1	0.01	−1.63	down
TC0100018126.hg.1	RP11-407H12.8	< 0.01	−1.57	down
TC1200010026.hg.1	RP11-1018 J8.2	0.01	1.56	up

The threshold for the differential expression of lncRNAs was set to a *P* value < 0.01

Group A consisted of patients with IMH; Group C consisted of patients with PDR

*IMH* Idiopathic macular hole, *PDR* Proliferative diabetic retinopathy

Meanwhile, RP4-631H13.2 expression levels in the vitreous of patients with PDR treated with anti-VEGF therapy were significantly higher than those in untreated patients with PDR, which is consistent with the trend of microarray profiling. The other lncRNAs, RP11-407H12.8, CTD-2532 K18.1, RP11-116 N8.4, and RP11-370P15.2, showed no significant differences (Fig. 5). However, no statistical differences were found in the plasma levels of these lncRNAs (Fig. 5 and S5).

## Discussion

The multifactorial pathogenesis of DR is not completely understood. As a new class of modulatory molecules, the essential roles of lncRNAs in the etiology of a broad spectrum of diseases have attracted considerable attention [6, 15, 16]. PDR is usually considered to be a neovascular disorder, and several lncRNAs have been implicated in neovascularization. ANRIL regulates VEGF expression and function in DR mediated by the PRC2 complex, thereby promoting new vessel formation [17]. MALAT1 promotes high glucose-induced human retinal endothelial cells (HRECs) by upregulating endoplasmic reticulum stress [18] or via suppressing the VE-cadherin/β-catenin complex by targeting miR-125b [19]. MIR497HG is downregulated after high-glucose treatment, and it suppresses the neovascularization of HRECs by targeting the miRNA-128-3p/SIRT axis [20]. SNHG7 inhibits high-glucose-induced angiogenesis by regulating the miR-543-mediated SIRT1/VEGF pathway [21]. TDRG1 promotes neovascularization by upregulating VEGF during DR [22]. Thus, dysregulated lncRNA expression is relevant to the molecular etiology of PDR.

Some researchers have focused on the expression of lncRNAs in DR using high-throughput screening technologies. Yan et al. first reported that approximately 303 lncRNAs are differentially expressed in the retinas of diabetic rats [7]. Likewise, to demonstrate the relationship between lncRNAs and anti-VEGF drugs, Wang et al. reported that 427 lncRNAs were differentially expressed after anti-VEGF treatment [23]. However, existing data are insufficient for the vitreous in PDR [9]. The transcriptional landscape of the vitreous, a reservoir of pathological signaling molecules, is a novel research field for PDR.

We analyzed the noncoding RNA transcript expression profiles of three patient groups: IMH, PDR treated with conbercept, and PDR alone. As a control for cytokine analyses in PDR, vitreous samples with idiopathic epiretinal membrane and/or IMH have been used in many studies [24–26]. Nonetheless, it has been reported that the activation conditions of inflammation and fibrosis in eyes with idiopathic epiretinal membranes should be carefully considered as a control group [27]. This study only included patients with IMH in the control group.

We identified that 1067 noncoding RNA transcripts were aberrantly expressed in patients with PDR. We also evaluated the effects of anti-VEGF therapy on the expression of lncRNA in patients with PDR. Ultimately, 835 dysregulated noncoding RNA transcripts were identified. We followed up on the differential expression analysis with qRT-PCR tests in a separate validation cohort, including six patients in group A, eight patients in group B, and twenty-five patients in group C. Most transcriptomes by qRT-PCR (6/10) were consistent with the results of gene microarray analysis, verifying the reliability of the microarray data.

Plasma samples from the same patient were subjected to qRT-PCR, which offers an alternative noninvasive strategy and helps to analyze whether a differential expression is more attributable to the local effect of the ocular vitreous or to the mutual influence of systemic and vitreoretinopathy modifications. The differential expression of all qRT-PCR-verified transcripts in plasma was not statistically significant, suggesting that these transcripts are likely to result from local differential expression of the ocular vitreous.

In this study, LINC01210 expression levels were significantly lower in the vitreous humor of patients with IMH patients in those study. LINC01210 is associated with the proliferative, migratory, and invasive abilities of cells [28, 29]. We need further investigation to verify the potential role of LINC01210 in PDR. To date, the effects of most lncRNAs tested by qRT-PCR in our study are not explicitly understood. In the future, comprehensive studies on the function of lncRNAs in the pathogenesis of PDR will help determine new and effective diagnostic and therapeutic targets.

Most lncRNAs were poorly annotated. Bioinformatics analysis was used to investigate further differentially expressed lncRNAs. Using bioinformatics analysis, we found that the mRNA expression levels of frizzled class receptor 6 (FZD6) and proteasomal subunit α4s (PSMA8), which associated with the Wnt signaling pathway and Alzheimer disease, were downregulated in the vitreous of patients with PDR. Of interest, increased Wnt signaling is one of the causes of pathological ocular neovascularization of DR [30, 31]. Further, multiple factors of DR have been shown to play a vital role in the development of neurodegeneration in Alzheimer’s disease [32]. These potential correlations will be explored in future studies; in vivo and in vitro studies should be performed to elucidate the molecular mechanisms of lncRNA-mediated PDR pathogenesis.

This study had certain limitations. First, the number of patients included in this study was relatively small. However, these results were statistically significant. We provided one of the few lncRNA expression profiles in individual vitreous samples of patients with PDR. Second, vitrectomy surgery for patients with DR was performed out of necessity, such as in cases of vitreous hemorrhage and retinal detachment [33]. Therefore, this study did not exclude patients with vitreous hemorrhage. If vitreous hemorrhage was incorporated, the surgeon avoided collecting as much blood as possible. Blood-borne molecules in vitreous samples cannot be completely eliminated [9]. Finally, a selection bias may exist. The effects of anti-VEGF drugs were evaluated in different cohorts because it is difficult to collect dissimilar vitreous samples from same patient with PDR.

## Conclusions

There were systemic expression differences in the vitreous between patients with PDR and those with IMH and between patients with PDR who had undergone anti-VEGF treatment and those who had not. The levels of RP11-573 J24.1, RP11-787B4.2, RP11-654G14.1, RP11-2A4.3, and RP11-502I4.3 in the vitreous humor of patients with PDR were lower than those in patients with IMH. RP4-631H13.2 in the vitreous of patients with PDR after anti-VEGF treatment showed increased expression compared to that in untreated patients with PDR.

## Supplementary Information

**Additional file 1: Table S1.** Primer sequence.

**Additional file 2: Fig. S1.** Heatmaps were generated from the hierarchical cluster analysis (Group B versus Group C).

**Additional file 3: Fig. S2.** GO Enrichment Analysis (Group B versus Group C).

**Additional file 4: Fig. S3.** KEGG Pathway Enrichment Analysis (Group B versus Group C).

**Additional file 5: Fig. S4.** The co-expression network of differential transcripts (Group B versus Group C).

**Additional file 6: Table S2.** Analysis of neighbor genes of the noncoding RNA transcripts (Group B versus Group C).

**Additional file 7: Table S3.** Dysregulated lncRNAs in microarray data analysis (Group B versus Group C).

**Additional file 8: Fig. S5.** Expression levels of lncRNAsof the confirmation cohort, in vitreous and plasma (Group B versus Group C).

## Data Availability

The datasets used during the current study are available from the corresponding author on reasonable request.

## Abbreviations

DR: Diabetic retinopathy
PDR: Proliferative diabetic retinopathy
VEGF: Vascular endothelial growth factor
lncRNAs: Long noncoding RNAs
mRNAs: Messenger RNAs
IMH: Idiopathic macular hole
TAC 4.0.2: Transcriptome Analysis Console 4.0.2
qRT-PCR: Quantitative real-time polymerase chain reaction
GO: Gene ontology
KEGG: Kyoto Encyclopedia of Genes and Genomes
GAPDH: Glyceraldehyde-3-phosphate dehydrogenase
FZD6: Frizzled class receptor 6
PSMA8: Proteasomal subunit α4s
