# Supplementary material for: Aberrant lncRNA expression in patients with proliferative diabetic retinopathy: preliminary results from a single-center observational study
Source: BMC Ophthalmol. 2023 Mar 10;23:94. doi: 10.1186/s12886-023-02817-4 (PMC9999565; doi:10.1186/s12886-023-02817-4)
Supplement: Supplementary file 1 — Additional file 1: Table S1. Primer sequence. [file 12886_2023_2817_MOESM1_ESM.docx]

**Table S1.** Primer sequence

| Primer name | Primer Sequences |
| --- | --- |
| RP11-573J24.1 | F: 5’- CAAGTGTGGGGCACTGTAGG - 3’ |
|  | R: 5’- TTGCAACCACTCCGCAGAA - 3’ |
| RP11-787B4.2 | F: 5’- AAACACGGGTCCAGATTGCT - 3’ |
|  | R: 5’- TCCTCGCAACAACTCCATGA - 3’ |
| RP11-654G14.1 | F: 5’- TGGAAGCTCCTGTCATCTGC - 3’ |
|  | R: 5’- GCACAACCACAAGGCGTTAC - 3’ |
| RP11-2A4.3 | F: 5’- AGCGCCTCAAAGCTTCCTAA - 3’ |
|  | R: 5’- GAGGGCTCACAATAAGCGGT - 3’ |
| RP11-502I4.3 | F: 5’- GAGACTGGTCTTGCTCGCTC - 3’ |
|  | R: 5’- ACAAAGTGGGGATAACCGGG - 3’ |
| RP11-407H12.8 | F: 5’- TCGAGAGCAGTGATGACCCT - 3’ |
|  | R: 5’- AAAGCTTTCAACCCCTCCCC - 3’ |
| RP11-116N8.4 | F: 5’- GCCAGCTTTGGAGATGAGTG - 3’ |
|  | R: 5’- GGCCTCCTGGATCCGTTACA - 3’ |
| RP11-370P15.2 | F: 5’- ATCACAGTTGACCAGGGGA - 3’ |
|  | R: 5’- AGGGCACATATCCTGACCAC - 3’ |
| RP4-631H13.2 | F: 5’- ATCACAGTTGACCAGGGGA - 3’ |
|  | R: 5’- AGGGCACATATCCTGACCAC - 3’ |
| CTD-2532K18.1 | F: 5’- CGAGGGCACGGATACAAATG - 3’ |
|  | R: 5’- TTGAATGAGGAGGTGCGTGG - 3’ |
| GAPDH | F: 5’- AAATTCCATGGCACCGTCAAG - 3’ |
|  | R: 5’- GGACTCCACGACGTACTCAG - 3’ |

qRT-PCR, quantitative real-time polymerase chain reaction; F, forward; R, reverse.
