# Supplementary material for: Aberrant lncRNA expression in patients with proliferative diabetic retinopathy: preliminary results from a single-center observational study
Source: BMC Ophthalmol. 2023 Mar 10;23:94. doi: 10.1186/s12886-023-02817-4 (PMC9999565; doi:10.1186/s12886-023-02817-4)
Supplement: Supplementary file 2 — Additional file 2: Fig. S1. Heatmaps were generated from the hierarchical cluster analysis (Group B versus Group C). [file 12886_2023_2817_MOESM2_ESM.pdf]

A

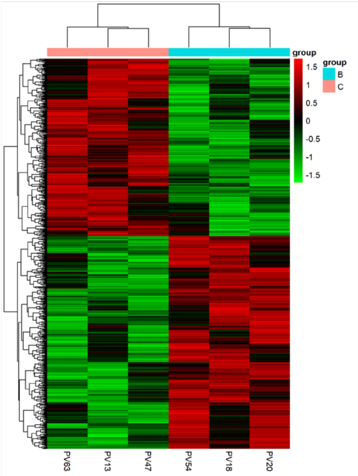

B

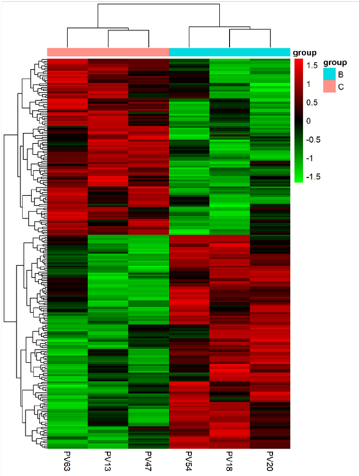

Figure S1. Heatmaps were generated from the hierarchical cluster analysis (Group B versus Group C). A: noncoding RNA transcripts; B: coding RNA transcripts. The color scale at the top illustrates the relative expression level of RNAs across all samples: red denotes expression greater than 0, and green denotes expression less than 0. Group B consisted of patients with PDR pretreated with conbercept 3–7 days before surgery; Group C consisted of patients with PDR who underwent surgery alone. PDR, proliferative diabetic retinopathy.
