# Supplementary material for: Aberrant lncRNA expression in patients with proliferative diabetic retinopathy: preliminary results from a single-center observational study
Source: BMC Ophthalmol. 2023 Mar 10;23:94. doi: 10.1186/s12886-023-02817-4 (PMC9999565; doi:10.1186/s12886-023-02817-4)
Supplement: Supplementary file 3 — Additional file 3: Fig. S2. GO Enrichment Analysis (Group B versus Group C). [file 12886_2023_2817_MOESM3_ESM.pdf]

A

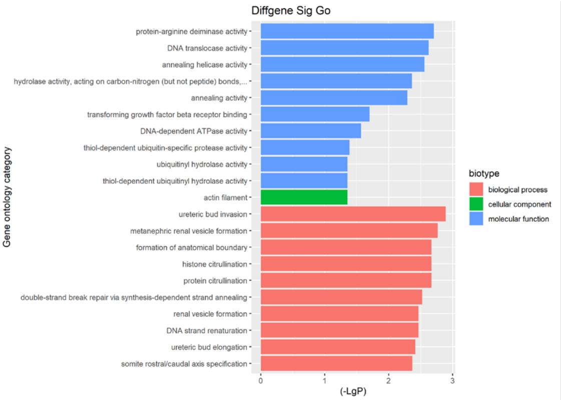

B

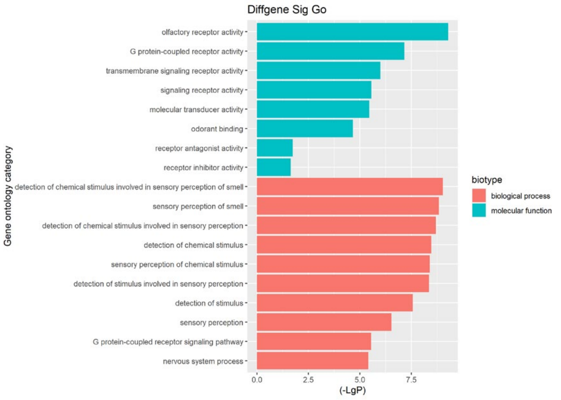

Figure S2. GO Enrichment Analysis (Group B versus Group C). A: upregulated transcripts; B: downregulated transcripts. The x-axis shows -log10 (P-value). The y-axis shows GO category. $P < 0.05$ was considered to be statistically significant. Group B consisted of patients with PDR pretreated with cobercept 3–7 days before surgery; Group C consisted of patients with PDR who underwent surgery alone. GO: Gene Ontology; PDR, proliferative diabetic retinopathy.
