# Supplementary material for: Aberrant lncRNA expression in patients with proliferative diabetic retinopathy: preliminary results from a single-center observational study
Source: BMC Ophthalmol. 2023 Mar 10;23:94. doi: 10.1186/s12886-023-02817-4 (PMC9999565; doi:10.1186/s12886-023-02817-4)
Supplement: Supplementary file 4 — Additional file 4: Fig. S3. KEGG Pathway Enrichment Analysis (Group B versus Group C). [file 12886_2023_2817_MOESM4_ESM.pdf]

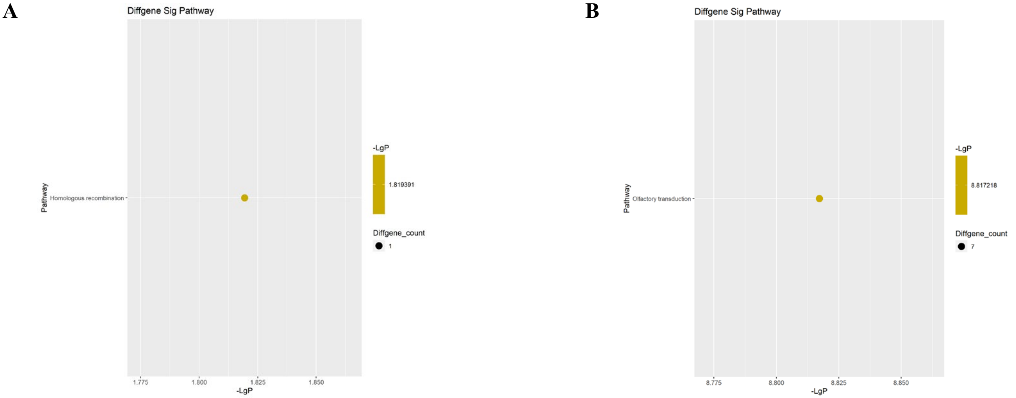

Figure S3. KEGG Pathway Enrichment Analysis (Group B versus Group C). As per the citation guidelines (www.kegg.jp/kegg/kegg1.html), KEGG analysis was used to predict the signaling pathways in which these differentially expressed transcripts may be involved [13-15]. These images were obtained by KEGG, and the Kanehisa laboratory have provided copyright permission. A: upregulated transcripts; B: downregulated transcripts. The x-axis shows -log10 (*P*-value). The y-axis shows enriched pathway. $P < 0.05$ was considered to be statistically significant. Group B consisted of patients with PDR pretreated with conbercept 3–7 days before surgery; Group C consisted of patients with PDR who underwent surgery alone. KEGG, Kyoto Encyclopedia of Genes and Genomes; PDR, proliferative diabetic retinopathy.
