# Supplementary material for: Aberrant lncRNA expression in patients with proliferative diabetic retinopathy: preliminary results from a single-center observational study
Source: BMC Ophthalmol. 2023 Mar 10;23:94. doi: 10.1186/s12886-023-02817-4 (PMC9999565; doi:10.1186/s12886-023-02817-4)
Supplement: Supplementary file 5 — Additional file 5: Fig. S4. The co-expression network of differential transcripts (Group B versus Group C). [file 12886_2023_2817_MOESM5_ESM.pdf]

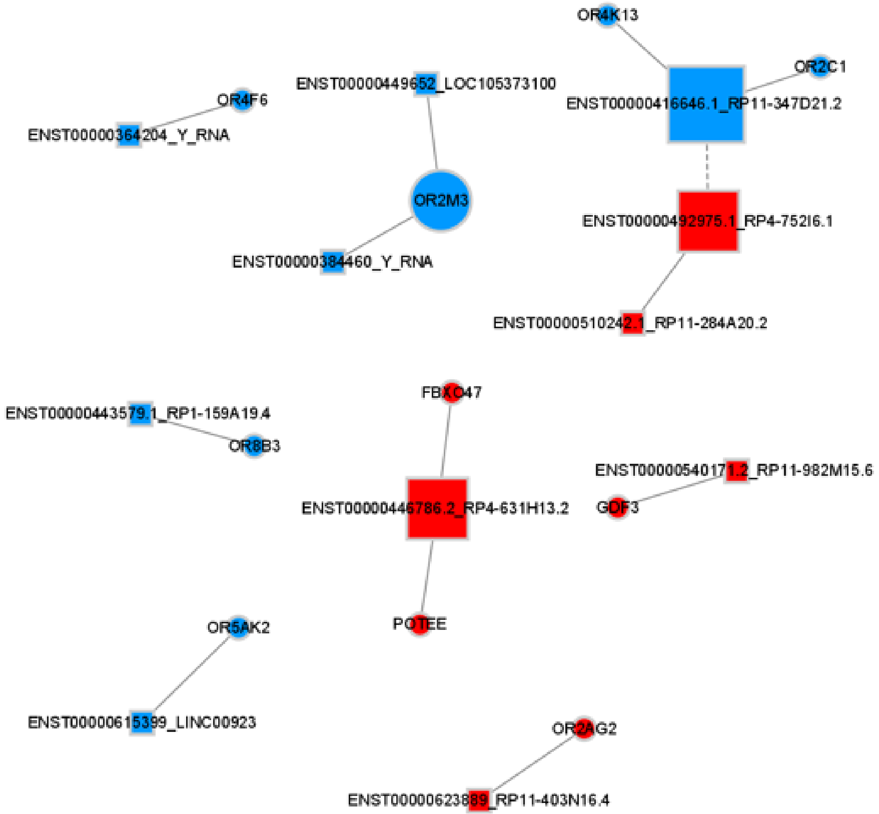

Figure S4. The co-expression network of differential transcripts (Group B versus Group C). Red squares indicate that noncoding RNA transcript expression is upregulated and blue squares indicate downregulation. Red circles indicate that coding RNA transcript expression is upregulated and blue circles indicate downregulation. $P < 0.05$ was considered to be statistically significant. Group B consisted of patients with PDR pretreated with conbercept 3–7 days before surgery; Group C consisted of patients with PDR who underwent surgery alone. PDR, proliferative diabetic retinopathy.
