# Supplementary material for: Aberrant lncRNA expression in patients with proliferative diabetic retinopathy: preliminary results from a single-center observational study
Source: BMC Ophthalmol. 2023 Mar 10;23:94. doi: 10.1186/s12886-023-02817-4 (PMC9999565; doi:10.1186/s12886-023-02817-4)
Supplement: Supplementary file 6 — Additional file 6: Table S2. Analysis of neighbor genes of the noncoding RNA transcripts (Group B versus Group C). [file 12886_2023_2817_MOESM6_ESM.docx]

**Table S2.** Analysis of neighbor genes of the noncoding RNA transcripts (Group B versus Group C)

| Gene Symbol | Fold Change | *P*-value | Regulation | Same Strand Gene with Overlap | Up Stream Gene in 10000bp | Down Stream Gene in 10000bp | Complementary Strand Gene with Overlap |
| --- | --- | --- | --- | --- | --- | --- | --- |
| AP001597.1 | 1.62 | 0.03 | up |  |  |  | CYYR1 |
| AC010082.2 | 1.78 | 0.03 | up |  |  |  | HDAC9 |
| CTA-747E2.8 | 1.71 | 0.03 | up | ZNRF3 |  |  |  |
| RP11-380G10.1 | 1.8 | 0.03 | up |  |  |  | ARPP21 |
| AC007036.4 | -1.95 | 0.03 | down | MTURN |  | MTURN |  |
| RP11-349J5.2 | 2 | 0.03 | up |  |  |  | ALDH8A1 |
| RP4-613B23.3 | -1.7 | 0.02 | down |  |  |  | HHATL |
| RP3-395P12.2 | -1.8 | 0.01 | down |  | TNFSF4 |  |  |
| CTA-282F2.4 | -1.53 | 0.04 | down | LARGE1 |  |  |  |
| RP4-631H13.2 | 1.62 | 0.01 | up |  |  |  | ZYG11A |
| RP4-752I6.1 | 1.87 | 0.02 | up |  |  |  | WASF2 |
| RP11-153M7.5 | 1.94 | 0.02 | up |  |  |  | RNF175 |
| RP11-364L4.3 | 1.75 | 0.01 | up |  |  | GAB1 |  |
| RP11-10A14.3 | 1.77 | 0.04 | up |  |  |  | PPP1R3B |
| RP11-360L9.7 | 2.29 | 0.01 | up |  |  |  | GINS4 |
| RP11-1018J8.2 | -1.62 | ＜0.01 | down | RERG | RERG |  |  |
| RP11-350G24.1 | 1.85 | 0.02 | up |  |  |  | ANO4 |
| RP11-310I24.1 | -1.54 | 0.01 | down |  |  |  | TMTC1 |
| RP11-1100L3.7 | -1.84 | 0.04 | down |  |  |  | ATG101 |
| RP11-722P11.4 | 1.83 | 0.04 | up |  |  |  | C12orf54 |
| RP11-638I2.9 | 1.58 | 0.03 | up |  | SLC25A47 |  | WARS |
| RP11-368P15.3 | -1.79 | 0.01 | down |  |  |  | DDHD1 |
| RP11-1042B17.3 | -1.69 | 0.01 | down |  | SIX6 |  | C14orf39 |
| RP11-973H7.1 | -1.97 | 0.01 | down |  | PTPN2 |  |  |
| RP11-327F22.5 | 1.75 | 0.02 | up |  | CYLD |  |  |
| CTC-510F12.6 | 1.65 | 0.02 | up | TSPAN16 | TSPAN16 |  | RAB3D |
| CTB-5E10.3 | 1.55 | 0.03 | up |  |  |  | MRI1 |

**Table S2.** Continued

| Gene Symbol | Fold Change | *P*-value | Regulation | Same Strand Gene with Overlap | Up Stream Gene in 10000bp | Down Stream Gene in 10000bp | Complementary Strand Gene with Overlap |
| --- | --- | --- | --- | --- | --- | --- | --- |
| CTD-2525I3.3 | -1.72 | 0.04 | down |  |  |  | PPP2R1A |
| RP11-727F15.11 | -1.76 | 0.02 | down |  | TMEM223 |  | TAF6L |
| RP11-362K14.7 | 2.01 | 0.01 | up |  | MYNN |  | LRRC34 |
| RP4-678D15.1 | -1.93 | 0.03 | down |  |  |  | TSHZ2 |
| RP11-803P9.1 | -1.56 | 0.03 | down |  | FYTTD1 | LRCH3 |  |

*P* < 0.05 was considered to be statistically significant.

Group B consisted of patients with PDR pretreated with conbercept 3–7 days before surgery; Group C consisted of patients with PDR who underwent surgery alone.

PDR, proliferative diabetic retinopathy.
