# Supplementary material for: Aberrant lncRNA expression in patients with proliferative diabetic retinopathy: preliminary results from a single-center observational study
Source: BMC Ophthalmol. 2023 Mar 10;23:94. doi: 10.1186/s12886-023-02817-4 (PMC9999565; doi:10.1186/s12886-023-02817-4)
Supplement: Supplementary file 7 — Additional file 7: Table S3. Dysregulated lncRNAs in microarray data analysis (Group B versus Group C). [file 12886_2023_2817_MOESM7_ESM.docx]

**Table S3.** Dysregulated lncRNAs in microarray data analysis (Group B versus Group C)

| Gene ID | Gene Symbol | *P*-value | Fold Change | Regulation |
| --- | --- | --- | --- | --- |
| TC0500009545.hg.1 | CTD-2532K18.1 | 0.01 | -2.26 | down |
| TC0100018126.hg.1 | RP11-407H12.8 | < 0.01 | 2.03 | up |
| TC0300009452.hg.1 | RP11-362K14.7 | 0.01 | 2.01 | up |
| TC1400008955.hg.1 | RP11-116N8.4 | 0.01 | 1.98 | up |
| TC0900009800.hg.1 | CTD-2532K18.1 | 0.01 | 1.89 | up |
| TC0600013130.hg.1 | RP1-86D1.3 | < 0.01 | -1.88 | down |
| TC1600007800.hg.1 | RP11-305A4.2 | < 0.01 | -1.82 | down |
| TC1600008091.hg.1 | RP11-370P15.2 | 0.01 | 1.81 | up |
| TC0100016430.hg.1 | RP3-395P12.2 | 0.01 | -1.80 | down |
| TC1400007183.hg.1 | RP11-368P15.3 | 0.01 | -1.79 | down |
| TC0400008877.hg.1 | RP11-364L4.3 | 0.01 | 1.75 | up |
| TC1200010026.hg.1 | RP11-1018J8.2 | < 0.01 | -1.62 | down |
| TC0100014213.hg.1 | RP4-631H13.2 | 0.01 | 1.62 | up |

The threshold for the differential expression of lncRNAs was set to a *P* value < 0.01.

Group B consisted of patients with PDR pretreated with conbercept 3–7 days before surgery; Group C consisted of patients with PDR who underwent surgery alone.

PDR, proliferative diabetic retinopathy.
