# Supplementary material for: Aberrant lncRNA expression in patients with proliferative diabetic retinopathy: preliminary results from a single-center observational study
Source: BMC Ophthalmol. 2023 Mar 10;23:94. doi: 10.1186/s12886-023-02817-4 (PMC9999565; doi:10.1186/s12886-023-02817-4)
Supplement: Supplementary file 8 — Additional file 8: Fig. S5. Expression levels of lncRNAsof the confirmation cohort, in vitreous and plasma (Group B versus Group C). [file 12886_2023_2817_MOESM8_ESM.pdf]

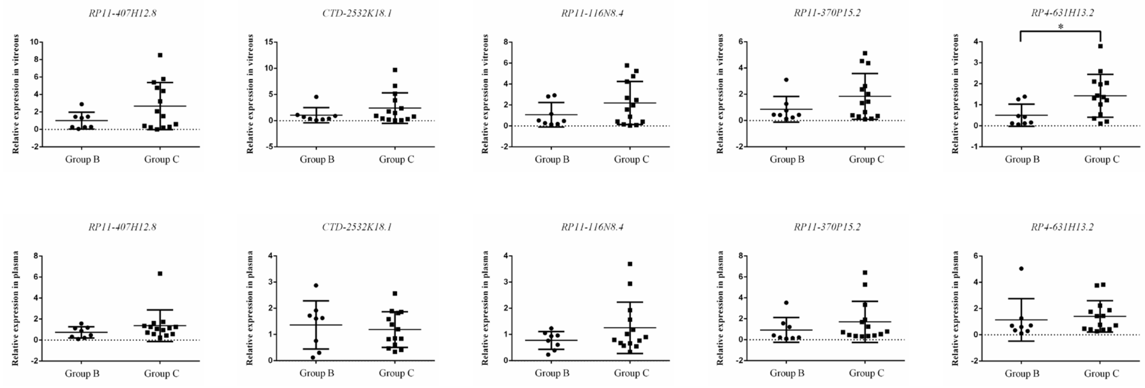

Figure S5. Expression levels of lncRNAs of the confirmation cohort, in vitreous and plasma (Group B versus Group C). The levels of lncRNAs were determined by qRT-PCR. The data of each group are expressed as the relative expression. The *asterisk* indicates the significant difference. Group B consisted of patients with PDR pretreated with conbercept 3–7 days before surgery; Group C consisted of patients with PDR who underwent surgery alone. qRT-PCR, quantitative real-time polymerase chain reaction; PDR, proliferative diabetic retinopathy.
